# Effects of curcumin supplementation on insomnia and daytime sleepiness in young women with premenstrual syndrome and dysmenorrhea: A randomized clinical trial 

**DOI:** 10.22038/AJP.2023.21916

**Published:** 2023

**Authors:** Saman Seyedabadi, Zahra Sadat Hoseini, Gordon A. Ferns, Afsane Bahrami

**Affiliations:** 1 *Cardiovascular Diseases Research Center, Birjand University of Medical Sciences, Birjand, Iran*; 2 *Department of Psychology, University of Birjand, Birjand, Iran*; 3 *Brighton & Sussex Medical School, Division of Medical Education, Falmer, Brighton, Sussex BN1 9PH, UK *; 4 *Clinical Research Development Unit, Imam Reza Hospital, Faculty of Medicine, Mashhad University of Medical Sciences, Mashhad, Iran*; 5 *Clinical Research Development Unit of Akbar Hospital, Faculty of Medicine, Mashhad University of Medical Sciences, Mashhad, Iran*

**Keywords:** Insomnia, Menstruation, Sleepiness, Turmeric

## Abstract

**Objective:**

Premenstrual syndrome and primary dysmenorrhea are common gynecological complaints that are associated with psychological disorders. There is increasing evidence for the neuroprotective properties of curcumin, a polyphenolic natural product. This study aimed to assess the effects of curcumin on sleep complications in women with premenstrual syndrome and dysmenorrhea.

**Materials and Methods:**

This triple-masked, placebo-controlled clinical trial comprised 124 patients with both premenstrual syndrome and dysmenorrhea. Participants were randomly assigned to curcumin (n=57) or control (n=60) groups. Each participant received one capsule containing either 500 mg of curcumin plus piperine or placebo, daily, from 7 days before until 3 days after menstruation for three consecutive menstrual cycles. Insomnia and sleepiness were assessed using standard questionnaires.

**Results:**

Scores for insomnia and daytime sleepiness were directly correlated with the Premenstrual Syndrome Screening Tool (PSST) score (p<0.05), but not with the visual analogue scale (VAS) score at baseline (p>0.05). There was a non-significant reduction in insomnia and sleepiness scores in both curcumin and placebo groups after the study intervention. Whilst, improvement rate of insomnia status, daytime sleepiness severity, short sleep duration and difficult sleep initiation was not statistically significant between the curcumin and placebo groups.

**Conclusion:**

Curcumin does not significantly affect sleep disorders in young women with premenstrual syndrome and dysmenorrhea.

## Introduction

A large proportion of women experience menstrual associated problems, such as dysmenorrhea and premenstrual syndrome (PMS) during their reproductive age (Bahrami et al., 2018c ▶). PMS refers to the cyclic cluster of physical, psychological and emotional symptoms initiating at the end of luteal phase alleviating several days after menstruation. Primary dysmenorrhea (PD) is characterized by severe abdominal cramping, sometimes headache and leg ache, as well as gastrointestinal symptoms which are caused by elevations of prostaglandin levels during menstruation (Bahrami et al., 2019 ▶). 

Menstrual complications have significant adverse effects on emotional states, and consequently interfere with quality of life. Increasing evidence indicates the connection between sleep quality and menstrual patterns, particularly the effect of menstrual associated symptoms on sleep health (Ayadilord et al., 2020 ▶; Erbil and Yücesoy, 2020 ▶; Nam et al., 2017 ▶; Wang et al., 2016). Recently, it has been reported that poor sleep quality, short sleep duration and insomnia are related to the severity of PMS (Xing et al., 2020 ▶). We have previously reported that PD women have a higher insomnia score, daytime dizziness and sleep apnea than those without it (Bahrami et al., 2017 ▶).

On the other hand, disturbed sleep pattern not only has a negative impact on cognitive abilities, emotional function, and other daytime functioning process, but also deeply affects hormonal regulation (Alger et al., 2014 ▶). Neuroendocrine disturbances induced by mental distress predominantly sleep deprivation affect the action of the hypothalamic-pituitary-adrenal axis and so influence the menstrual cycle (Dorn et al., 2009 ▶; Yu et al., 2017 ▶). 

There are few therapeutic options for the relief of PD or PMS symptoms. Psychological, anovulatory cycles, supplements, traditional and non-pharmacological medicines have been used in the management of these symptoms (Bahrami et al., 2018b ▶; Maharaj and Trevino, 2015 ▶; Vaghela et al., 2019 ▶). Due to the long-term side effects and costs of chemical agents, attention has been focused on the treatment through alternative medicines in recent years. Complementary and herbal medicines are widely used as an alternative to prescription medicines in the treatment of many medical situations i.e. PMS, menopausal signs and PD (Whelan et al., 2009 ▶). 

Curcumin is the yellow active ingredient of the rhizomes of *Curcuma longa* which is extensively used as herbal medicine in different countries (Bahrami et al., 2018a ▶; Parsamanesh et al., 2018 ▶). Curcumin is well known for its potent biologic and pharmacologic effects especially antioxidant, anti-inflammatory, antitumoral, immunomodulatory (Hatamipour et al., 2018 ▶), anti-aging (Abrahams et al., 2019 ▶), and neuroprotective (Motaghinejad et al., 2017 ▶) properties. 

Although curcumin has been studied in diverse clinical settings, clinical trials assessing its effect on sleep-associated complaints are scarce and have been inconsistent (Maghbooli et al., 2019 ▶; Saberi-Karimian et al., 2021 ▶). As yet most of the investigations have evaluated the effects of curcumin on sleep in experimental animals. Curcumin prevents neuronal loss, memory impairment as well as structural and behavioral changes caused by chronic sleep deprivation in the rat brain by reducing oxidative stress (Erfanizadeh et al., 2020 ▶; Noorafshan et al., 2017a ▶; Noorafshan et al., 2017b ▶).

Regarding the significant reported therapeutic effects of curcumin in previous studies and, the potential of using it as a cost-effective and available natural product, this study aimed to examine its potential as a natural compound for alleviation of psychological aspect of gynecological disorders especially sleep complications. Therefore, because of the pleiotropic activities of curcumin such as its anti-inflammatory and antioxidant effects, we assessed the effects of curcumin supplement on sleep disorders in young women with PD and PMS.

## Materials and Methods

**Study design**

This randomized, triple-blind, placebo-controlled trial, was approved by the Ethnic committee of Birjand University of Medical Sciences code: IR.BUMS.REC.1398.160), and registered at Iranian Registry of Clinical Trial (Trial ID: IRCT20191112045424N1 on 23 January 2020; available at https://www.irct.ir). The study participants comprised 124 female students who lived in 4 distinct university dormitories in Birjand, in South-Eastern of Iran, from January 2020 to April 2020. Researcher, data collectors, patients, and statistical analysts were blinded to the group allocations of participants. and all participants signed written informed consent for study participation. 

The sample size was calculated using α= 0.05, β=0.2 and with a 95% confidence interval using a power calculation; it was estimated that at least 55 patients were needed for each arm, and the final sample size assuming drop-out rate was set as 62 patients in each group (Ghayour Mobarhan et al., 2020 ▶).

$n=\frac{{{(z}_{1-\frac{\alpha }{2}}+z_{1-\beta })}^{2}(S_{1}^{2}+S_{2}^{2})}{({\overset{\text{®}}{x_{1}}-{\overset{\text{®}}{x}}_{2})}^{2}}$

The inclusion criteria were age 18-24 years, being single, having a negative history of gynecological disorders and any sensitivity to herbal agents, having menses on a regular base, with having both moderate to intense PD and PMS. Women who had any acute or chronic illness or were on any medications, were married or experienced stressful events during the intervention period, were excluded.

PD and PMS were diagnosed by a gynecologist based on the results of the visual analogue scale (VAS) (Crichton, 2001 ▶) and Premenstrual Syndrome Screening Tool (PSST) (Steiner et al., 2003 ▶), respectively as described previously (Ayadilord et al., 2020 ▶). 

Women who complied with the inclusion criteria and agreed to trial participation, were registered to the study and were randomized to the two groups. Masking of the group allocation was continued until the final analyses were performed and all was conducted by a specialist nurse at the clinic.

**Intervention**

Participants were randomly allocated (1:1 ratio) to the curcumin or control group and assigned to take the curcumin (n=62) or placebo (n=62). Curcumin group received (Curcumin plus 5 mg piperine; C3 Complex, supplied by Sami Labs Ltd, Bangalore, India) at a daily dose of 500 mg (Anand et al., 2007 ▶). The placebo capsules contained inert particles (500 mg lactose powder plus 5 mg piperine; Sami Labs Ltd, Bangalore, India). Curcumin and placebo capsules were coded as “A” or “B” by the pharmacy, were indistinguishable on appearance, size, and color and all investigators participated in the study were blinded to the allocation. A statistician prepared a randomized list using NCSS (statistical software) by the simple block randomization method regarding to CONSORT guidelines. The participants were given one capsule per day for 10 days (7 days before and until 3 days after beginning of menstrual period) for 3 menstrual periods. Compliance and any probable side effects were followed during and after study in both groups. Treatment compliance was evaluated through volunteer-reported pill count and telephone follow-up. 

**Insomnia assessment**

A valid and reliable Persian version of Insomnia Severity Index (ISI) as a self-report instrument which measures severity of insomnia based on patient’s own perception was used in this study (Yazdi et al., 2012 ▶). Each item is rated on a zero to 4 point scale to provide a total score ranging from 0 to 28. Higher scores indicate a greater degree of insomnia (Ayadilord et al., 2020 ▶; Bastien et al., 2001 ▶).

**Daytime sleepiness assessment**

The Persian version of Epworth Sleepiness Scale (ESS) was employed to evaluate the participant's daytime sleepiness severity (Haghighi et al., 2013 ▶; Johns, 1993 ▶). This instructs participants to score their sleepiness during eight daily conditions from 0 to 3. The final scores range between 0 (no) to 24 (excessive daytime sleepiness). 

**Short sleep duration and difficult sleep initiating**

Short sleep duration as well as difficult sleep initiating were assessed based on two questions associated with sleep during the previous month: (1) “Do you experience difficulty falling asleep at nights?” (2) “How often have you woken up hurried and have problem going back to sleep? Sleep duration was measured using this question: “how many hours of real nocturnal sleep do you get on weekdays? Short sleep duration was considered if a person slept < 5 hr/day once or more weekly. Difficult sleep beginning was set as having difficulties falling asleep in 30 min once or more during a week (Cappuccio et al., 2008 ▶; Dj et al., 1989 ▶; Doi et al., 2000 ▶; Kurotani et al., 2015 ▶).

**Statistical analyses**

Statistical analysis was undertaken using SPSS 16 software. Kolmogorov–Smirnov test was applied to assess the normality of data distribution. Descriptive data are expressed as mean±SD or median (interquartile range) or number (percent). Correlation between variables was assessed using Pearson or Spearman tests.

Levene’s test for the homogeneity of variances between the groups for all measures demonstrated that the variances were identical (p>0.05) for all variables. Independent sample T-tests or Mann-Whitney test or Chi-square test were done to assess the differences in variables at baseline. Improvement rate is defined as number (percent) of participants whose “Moderate/Severe symptoms” improved to “Mild symptom” or from “Mild symptom” to “No problem status” or from “Moderate/Severe symptoms” to “No problem status”. The significance of changes from pre- to post-intervention within the group was investigated using paired T-tests or Wilcoxon Signed-Ranks Test. The alpha level set significant at p value less than 0.05 in all analyses. ANCOVA test was applied to disclose any differences in two intervention groups at the end of trial with adjusting for baseline values. 

## Results

**Figure 1:**
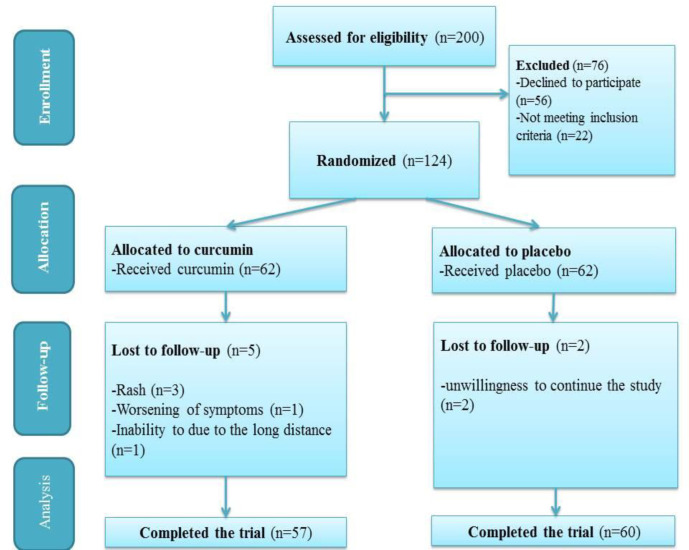
CONSORT flow diagram of trial

Out of one hundred and twenty-four participants who entered the trial, 117 patients completed the follow-up (n=57 in curcumin group and n=60 in placebo group) and were included in the final analyses. Seven participants did not complete the study (Figure 1). 

**Figure 2:**
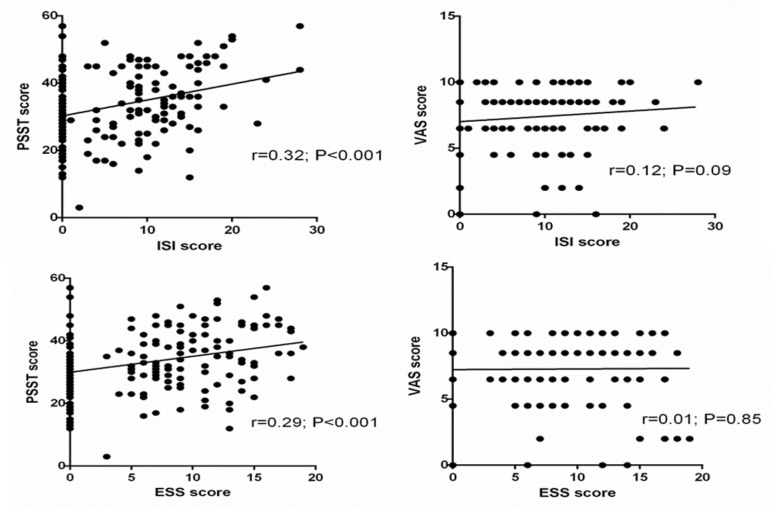
Correlation coefficient between Insomnia Severity Index (ISI) and Epworth Sleepiness Scale (ESS) scores with PSST and VAS scores at baseline

At baseline, the total score of insomnia and daytime sleepiness in this population was significantly correlated with the PSST score (r=0.32, p<0.001; r=0.29, p<0.001, respectively), but not with the VAS score (r=0.12, p=0.09; r=0.01, p=0.85, respectively; Figure 2). 

There were no significant differences between the curcumin and placebo groups with respect to the age and BMI at baseline (p>0.05). Table 1 demonstrates the main parameters before and after intervention in the curcumin and placebo groups. No significant differences were detected between the two allocation groups corresponding to insomnia score, daytime sleepiness score, and nocturnal sleep hour at baseline (p>0.05). 

Within-group analysis showed a non-significant reduction in insomnia score and sleepiness score (p>0.05), but significant increments in nocturnal sleep hour after curcumin supplementation (p<0.001).

The insomnia score and sleepiness remained unaltered by the end of study, although nocturnal sleep hour significantly increased (p<0.001) in the placebo group. At the end of the follow-up, results of ANCOVA test using baseline values as covariates showed no statistically significant difference in the reduction of insomnia and sleepiness scores as well as increment in nocturnal sleep hour between the two arms (p>0.05; Table 1).

However, the improvement rate regarding to insomnia status, daytime sleepiness severity, short sleep duration and difficult sleep initiation was not statistically significant between the curcumin and placebo groups (p>0.05; Table 2).

A significant decrement was found in PSST score after the intervention in the curcumin (32.1±9.6 to 20.4±9.8, p<0.001; net changes: -11.7±13.4) and placebo groups (30.9±8.2 to 22.2±9.9, p<0.001; net changes: 8.6±9.7). There was no significant spearman’s correlation between scores of ΔISI with ΔPSST and ΔVAS scores in either curcumin (r=-0.08, p=0.55; and r=-0.04, p=0.78, respectively) or placebo groups (r=0.1, p=0.45; and r=0.21, p=0.10, respectively). ΔESS score also was not correlated with ΔPSST and ΔVAS scores in the curcumin group (r=0.03, p=0.80; and r=-0.01, p=0.95, respectively) or the placebo group (r=-0.08, p=0.55; and r=-0.12, p=0.36, respectively).

**Table 1:** Comparison of main measures in the treatment groups before and after intervention

**Variables**	**Measurement period**	**Curcumin group**	**Placebo group**	**p** ^a^
Insomnia (score)	Before intervention	4.0(0.25-11.0)	6.0(0.25-11.5)	0.79
	After intervention	0.0(0.0-7.25)	2.0(0.0-8.5)	0.71
	p^b^	0.072	0.054	
Daytime sleepiness (score)	Before intervention	7.0(0.25-11.0)	7.0(0.25-11.0)	0.95
	After intervention	0.0(0.0-9.0)	0.0(0.0-8.0)	0.83
	p^b^	0.15	0.10	
Nocturnal sleep (hour)	Before intervention	7.2±1.2	7.2±1.4	0.80
	After intervention	8.2±1.4	8.0±1.4	0.62
	p^b^	<0.001	<0.001	

Values expressed as mean±SD (normally distributed variables) or median and interquartile range (non-normally distributed variables).^a^ p values indicate comparison between the groups by using independent sample t test (normally distributed variables) or Mann-Whitney (non-normally distributed variables) at baseline and ANCOVA test after treatment.^b ^p values indicate comparison within the groups by using paired-sample T test (normally distributed variables) or Wilcoxon test (non-normally distributed variables)

**Table 2:** Effect of curcumin vs. placebo on the sleep complications improvement

Variables		Curcumin group				Placebo group					
		Before intervention	After intervention	Improved	p value ^a^	Before intervention	After intervention	Improved	p value^ a^	p value
Insomnia status	No	36(63.2)	43 (75.4)	26.3%	0.15	33(55.0)	42(70.0)	31.0%	0.055	0.77^ b^
	Mild	15(26.3)	10(17.5)			18(30.0)	15(25.0)			
	Moderate/Severe	6(10.5)	4(7.0)			9(15.0)	3(5.0)			
Daytime sleepiness severity	No	39(68.4)	46(80.7)	24.6%	0.08	41(68.3)	47(78.3)	18.3%	0.14	0.52^ b^
	Mild/Moderate	15(26.3)	10(17.5)			15(25.0)	10(16.7)			
	Severe	3(5.3)	1(1.8)			4(6.7)	3(5.0)			
Short sleep duration	Yes	3 (5.3)	2(3.5)	33%	0.92	7 (11.7)	3(5.0)	42%	0.41	0.63^ b^
Difficult sleep initiation	Yes	16(28.1)	15(26.3)	6%	0.87	23(38.3)	19(31.7)	13.0%	0.21	0.73^ b^

Data presented as number (percent)^a^ Comparison of before vs. after values in each group (Wilcoxon test) ^b^ Comparison of improvement rates between the groups (Chi-square test)

## Discussion

This is the first clinical trial evaluating whether curcumin intervention can affect sleep-related complications in women suffering from PD and PMS. In the present study, after 3 consecutive menstrual cycle of treatment with curcumin, insomnia status, daytime sleepiness severity, short sleep duration, and difficult sleep initiation were not affected in patients with both PMS and PD. 

Insomnia and sleepiness are two common sleep disorders that affect a large percentage of the world’s population (Morin et al., 2006 ▶; Young, 2004 ▶). Insomnia is the most prevalent sleep-related complaint which is defined by waking up in the middle of the night and having trouble maintaining asleep (Pallesen et al., 2014 ▶). Daytime sleepiness, defined as a reduced capacity in maintaining a desirable level of wakefulness, has adverse consequence on individual’s health and quality of life (Young, 2004 ▶). We found that the total score for insomnia and daytime sleepiness was positively correlated with severity of PMS symptoms as illustrated by PSST score. Consistent with our findings, in two other studies done among nursing and medical students, premenstrual syndrome scale score was positively correlated with the Pittsburgh sleep quality index score (Aşcı et al., 2015 ▶; Erbil and Yücesoy, 2020 ▶). The menstrual phase is found to affect stage 2 sleep and REM sleep (Shechter et al., 2012 ▶). PMS women had lower response to melatonin in luteal phase of menstrual cycle versus the follicular phase; consequently deregulation of circadian rhythm alteration is responsible for the development of the psychological distress at the ending of luteal phase (Parry et al., 1996 ▶). Furthermore, higher levels of progesterone and lower levels of its metabolite, allopregnanolone, promote sleep complications in the luteal phase. In PMS women, in the luteal phase the amount of allopregnanolone decreases even further, which leads to elevated concentrations of gamma aminobutyric acid (GABA) because of an incapability to increase GABA mediated inhibition. Higher amounts of GABA possibly cause sleep disruptions in PMS women (Baker and Driver, 2007 ▶).

There is little evidence on the effect of curcumin administration on PMS and PD symptoms. Dyawapur et al*.* have expressed that curcumin supplementation significantly reduce PD pain in adolescent girls (Dyawapur et al., 2018 ▶). In a study performed by Khayat et al. intervention with 200 mg/day curcumin alleviated the physical, behavioral and somatic symptoms of PMS in these patients (Khayat et al., 2015 ▶). Also, curcumin could improve the psychological-associated problems of this syndrome, through increasing the serum levels of brain-derived neurotropic factor in cases with PMS (Fanaei et al., 2016 ▶).

This investigation showed that curcumin supplementation has no remarkable effect on the total grade of insomnia, daytime sleepiness severity, short sleep duration, and difficult sleep initiation. Recently, Saberi-Karimian et al*.* reported that curcumin does not influence sleep duration in individuals with metabolic syndrome (Saberi-Karimian et al., 2021 ▶). Moreover, curcumin supplementation 200 mg/day for 2 months did not improve insomnia in patients with osteoarthritis (Belcaro et al., 2010 ▶). But other relevant studies showed that curcumin intake significantly improved the quality of sleep in patients with Parkinson’s disease (Maghbooli et al., 2019 ▶), type 2 diabetes, and cardiovascular diseases (Shafabakhsh et al., 2020a ▶). This discrepancy might be because of the various study designs and patients, dosage and form of curcumin as well as length of the intervention.

We observed significant increments in nocturnal sleep hour in both curcumin and placebo allocations. A so-named ‘placebo effect’ (mental response which improves symptoms) might account for this finding (De Craen et al., 1999 ▶). Piperine is an alkaloid which was added to the curcuminoids to enhance its oral bioavailability and enteric absorbency (Shoba₁ et al., 1998 ▶). Additionally, our placebo capsules also contained piperine as a bioactive alkaloid with possible other additional favorable effects such as anti-inflammatory and anti-nociceptive characteristics (Bang et al., 2009 ▶; Srinivasan, 2007 ▶). Thus, these effects could also be reasons for nocturnal sleep duration increment in the placebo group.

Oxidative stress and inflammation contribute to the etiology of menstrual pain and associated symptoms (Bahrami et al., 2020 ▶; Frankel et al., 2021 ▶; Szmidt et al., 2020 ▶), and they have been found to be complicated in sleep, while their definite effects are not understood yet (Gozal, 2009 ▶; Mills et al., 2007 ▶). PMS and PD are risk factors for insufficient sleep. Also, the risk of menstrual associated symptoms is high in patients with sleep deprivation (Nam et al., 2017 ▶). Recently, positive association was reported between chronic inflammation and sleep disorders, indicating their aggravating relationship (Aricioglu and Cetin, 2020 ▶; Irwin, 2019 ▶). It has been suggested that antioxidants such as curcumin can improve symptoms of sleep disturbance by scavenging free radicals and thus mitigating inflammation (Shafabakhsh et al., 2020b ▶). Moreover, the curcumin's antidepressant action has been shown to be due to regulating the release of dopamine and serotonin (Kulkarni and Dhir, 2010 ▶; Lopresti et al., 2012 ▶). Serotonin participates in the control of mood, sleep, cognitive abilities and sexual function in women (Martinowich and Lu, 2008). Serotonin is also implicated in pathoetiology of PMS, particularly in initiation of mood and psychological manifestations (Marjoribanks et al., 2013 ▶; Wichianpitaya and Taneepanichskul, 2013 ▶). 

This was a sub-study of our previous triple-blinded controlled trial on curcumin’s effects on menstrual-associated symptoms in women with PMS and PD (Bahrami et al., 2021 ▶). Curcumin was safe and well tolerated in the current clinical trial. There was no report of severe side effects. There were only three cases reporting a rash and one case with worsening of PMS symptoms. This investigation was rigorously carried out and the strengths of the present study included its novelty, strict inclusion criteria, the use of a curcumin formulation with improved bioavailability, and a triple‐blind, placebo‐controlled design. But this study has several limitations. First, self-report questionnaires were applied to detect changes in sleep-related symptoms. Whereas this provides valid indices of clinical progress, such as polysomnography as reliable device, will support a more potent assessment of the clinical efficacy of curcumin. Second, it seems that 3 successive menstrual cycle supplementation of curcumin is not adequate time to affect the parameters evaluated in this trial. Moreover, we did not provide evidence about any interference or synergic effect between curcumin and piperine. Finally, the restricted age range and marital status abrogate generalizability of our findings to all premenopausal and/or married women with these characteristics.

The most notable finding of the present study was that supplementation with 500 mg/day curcumin did not attenuate the severity of insomnia, sleepiness, or other sleep-related problems in women suffering from PD and PMS. Larger and long-duration clinical trials with differing dosage of curcumin are needed to provide more comprehensive data regarding curcumin intervention and sleep. 

## Conflicts of interest

The authors have declared that there is no conflict of interest. We also declared no financial or other conflict with Sami Labs Ltd, Bangalore, India.

## References

[B1] Abrahams S, Haylett WL, Johnson G, Carr JA, Bardien S (2019). Antioxidant effects of curcumin in models of neurodegeneration, aging, oxidative and nitrosative stress: a review. Neuroscience.

[B2] Alger SE, Chambers AM, Cunningham T, Payne JD (2014). The role of sleep in human declarative memory consolidation. Curr Top Behav Neurosci.

[B3] Anand P, Kunnumakkara AB, Newman RA, Aggarwal BB (2007). Bioavailability of curcumin: problems and promises. Mol Pharm.

[B4] Aricioglu F, Cetin M (2020). A quadruple relationship: sleep, immune system, inflammation and psychiatric disorders. Psychiatry Clin Psychopharmacol.

[B5] Aşcı Ö, Gökdemir F, Süt HK, Payam F (2015). The relationship of premenstrual syndrome symptoms with menstrual attitude and sleep quality in Turkish nursing student. J Caring Sci.

[B6] Ayadilord M, Mahmoudzadeh S, Hoseini ZS, Askari M, Rezapour H, Saharkhiz M, Abbaszadeh A, Karbasi S, Dashtebayaze NZ, Ferns GA (2020). Neuropsychological function is related to irritable bowel syndrome in women with premenstrual syndrome and dysmenorrhea. Arch Gynecol Obstet.

[B7] Bahrami A, Atkin SL, Majeed M, Sahebkar A (2018a). Effects of curcumin on hypoxia-inducible factor as a new therapeutic target. Pharmacol Res.

[B8] Bahrami A, Avan A, Sadeghnia HR, Esmaeili H, Tayefi M, Ghasemi F, Nejati Salehkhani F, Arabpour-Dahoue M, Rastgar-Moghadam A, Ferns GA (2018b). High dose vitamin D supplementation can improve menstrual problems, dysmenorrhea, and premenstrual syndrome in adolescents. Gynecol Endocrinol.

[B9] Bahrami A, Bahrami-Taghanaki H, Khorasanchi Z, Timar A, Jaberi N, Azaryan E, Tayefi M, Ferns GA, Sadeghnia HR, Ghayour-Mobarhan M (2020). Menstrual problems in adolescence: relationship to serum vitamins A and E, and systemic inflammation. Arch Gynecol Obstet.

[B10] Bahrami A, Gonoodi K, Khayyatzadeh SS, Tayefi M, Darroudi S, Bahrami-Taghanaki H, Eslami S, Jaberi N, Ferns GA, Farahmand K (2019). The association of trace elements with premenstrual syndrome, dysmenorrhea and irritable bowel syndrome in adolescents. Eur J Obstet Gynecol Reprod Biol.

[B11] Bahrami A, Mazloum SR, Maghsoudi S, Soleimani D, Khayyatzadeh, SS, Arekhi S, Arya A, Mirmoosavi SJ, Ferns GA, Bahrami-Taghanaki H, Ghayour-Mobarhan M (2018c). High dose vitamin D supplementation is associated with a reduction in depression score among adolescent girls: a nine-week follow-up study. J Diet Suppl.

[B12] Bahrami A, Sadeghnia H, Avan A, Mirmousavi SJ, Moslem A, Eslami S, Heshmati M, Bahrami-Taghanaki H, Ferns GA, Ghayour-Mobarhan M (2017). Neuropsychological function in relation to dysmenorrhea in adolescents. Eur J Obstet Gynecol Reprod Biol.

[B13] Bahrami A, Zarban A, Rezapour H, Agha Amini Fashami A, Ferns GA (2021). Effects of curcumin on menstrual pattern, premenstrual syndrome, and dysmenorrhea: A triple‐blind, placebo‐controlled clinical trial fects of curcumin on menstrual pattern, premenstrual syndrome, and dysmenorrhea: A triple‐blind, placebo‐controlled clinical trial. Phytother Res.

[B14] Baker FC, Driver HS (2007). Circadian rhythms, sleep, and the menstrual cycle. Sleep Med.

[B15] Bang JS, Choi HM, Sur BJ, Lim SJ, Kim JY, Yang HI, Yoo MC, Hahm DH, Kim KS (2009). Anti-inflammatory and antiarthritic effects of piperine in human interleukin 1β-stimulated fibroblast-like synoviocytes and in rat arthritis models. Arthritis Res Ther.

[B16] Bastien CH, Vallières A, Morin CM (2001). Validation of the insomnia severity index as an outcome measure for insomnia research. Sleep Med.

[B17] Belcaro G, Cesarone M, Dugall M, Pellegrini L, Ledda A, Grossi M, Togni S, Appendino G (2010). Product-evaluation registry of Meriva®, a curcumin-phosphatidylcholine complex, for the complementary management of osteoarthritis. Panminerva Med.

[B18] Cappuccio FP, Taggart FM, Kandala NB, Currie A, Peile E, Stranges S, Miller MA (2008). Meta-analysis of short sleep duration and obesity in children and adults. Sleep.

[B19] Crichton N (2001). Visual analogue scale (VAS). J Clin Nurs.

[B20] De Craen AJ, Kaptchuk TJ, Tijssen JG, Kleijnen J (1999). Placebos and placebo effects in medicine: historical overview. J R Soc Med.

[B21] Dj B, Reynolds C, Monk T, Berman S, Kupfer D (1989). The Pittsburgh Sleep Quality Index: a new instrument for psychiatric practice and research. Psychiatry Res.

[B22] Doi Y, Minowa M, Uchiyama M, Okawa M, Kim K, Shibui K, Kamei Y (2000). Psychometric assessment of subjective sleep quality using the Japanese version of the Pittsburgh Sleep Quality Index (PSQI-J) in psychiatric disordered and control subjects. Psychiatry Res.

[B23] Dorn LD, Negriff S, Huang B, Pabst S, Hillman J, Braverman P, Susman EJ (2009). Menstrual symptoms in adolescent girls: association with smoking, depressive symptoms, and anxiety. J Adolesc Health.

[B24] Dyawapur A, Patil NG, Metri L (2018). Effectiveness of cinnamon tea and turmeric water for reducing dysmenorrhoea among degree girls. IJSHR.

[B25] Erbil N, Yücesoy H (2020). Relationship between premenstrual syndrome and sleep quality among nursing and medical students. Perspect Psychiatr Care.

[B26] Erfanizadeh M, Noorafshan A, Namavar MR, Karbalay-Doust S, Talaei-Khozani T (2020). Curcumin prevents neuronal loss and structural changes in the superior cervical (sympathetic) ganglion induced by chronic sleep deprivation, in the rat model. Biol Res.

[B27] Fanaei H, Khayat S, Kasaeian A, Javadimehr M (2016). Effect of curcumin on serum brain-derived neurotrophic factor levels in women with premenstrual syndrome: A randomized, double-blind, placebo-controlled trial. Neuropeptides.

[B28] Frankel RA, Michels KA, Kim K, Kuhr DL, Omosigho UR, Wactawski-Wende J, Levine L, Perkins NJ, Mumford SL (2021). Serum antioxidant vitamin concentrations and oxidative stress markers associated with symptoms and severity of premenstrual syndrome: a prospective cohort study. BMC Women's Health.

[B29] Ghayour Mobarhan M, Saberi-Karimian M, Qazizadeh H, Mohammadzadeh E, AA Ferns G, Sahebkar AH (2021). Does curcumin have an effect on sleep duration in metabolic syndrome patients?. Avicenna J Phytomed.

[B30] Gozal D (2009). Sleep, sleep disorders and inflammation in children. Sleep Med.

[B31] Haghighi KS, Montazeri A, Mehrizi AK, Aminian O, Golkhandan AR, Saraei M, Sedaghat M (2013). The Epworth Sleepiness Scale: translation and validation study of the Iranian version. Sleep Breath.

[B32] Hatamipour M, Johnston TP, Sahebkar A (2018). One molecule, many targets and numerous effects: the pleiotropy of curcumin lies in its chemical structure. Curr Pharm Des.

[B33] Irwin MR (2019). Sleep and inflammation: partners in sickness and in health. Nat Rev Immunol.

[B34] Johns MW (1993). Daytime sleepiness, snoring, and obstructive sleep apnea: the Epworth Sleepiness Scale. Chest.

[B35] Khayat S, Fanaei H, Kheirkhah M, Moghadam ZB, Kasaeian A, Javadimehr M (2015). Curcumin attenuates severity of premenstrual syndrome symptoms: A randomized, double-blind, placebo-controlled trial. Complement Ther Med.

[B36] Kulkarni S, Dhir A (2010). An overview of curcumin in neurological disorders. Indian J Pharm Sci.

[B37] Kurotani K, Kochi T, Nanri A, Eguchi M, Kuwahara K, Tsuruoka H, Akter S, Ito R, Pham NM, Kabe I (2015). Dietary patterns and sleep symptoms in Japanese workers: the Furukawa Nutrition and Health Study. Sleep Med.

[B38] Lopresti AL, Hood SD, Drummond PD (2012). Multiple antidepressant potential modes of action of curcumin: a review of its anti-inflammatory, monoaminergic, antioxidant, immune-modulating and neuroprotective effects. J Psychopharmacol.

[B39] Maghbooli M, Safarnejad B, Mostafavi H, Mazloomzadeh S, Ghoreishi A (2019). Effect of nanomicelle curcumin on quality of life and sleep in patients with parkinson’s disease: a double-blind, randomized, and placebo-controlled trial. Int Clin Neurosci J.

[B40] Maharaj S, Trevino K (2015). A comprehensive review of treatment options for premenstrual syndrome and premenstrual dysphoric disorder. J Psychiatr Pract.

[B41] Marjoribanks J, Brown J, O'Brien PMS, Wyatt K (2013). Selective serotonin reuptake inhibitors for premenstrual syndrome. Cochrane Database Syst Rev.

[B42] Martinowich K, Lu B (2008). Interaction between BDNF and serotonin: role in mood disorders. Neuropsychopharmacology.

[B43] Mills PJ, von Känel R, Norman D, Natarajan L, Ziegler MG, Dimsdale JE (2007). Inflammation and sleep in healthy individuals. Sleep.

[B44] Morin CM, LeBlanc M, Daley M, Gregoire J, Merette C (2006). Epidemiology of insomnia: prevalence, self-help treatments, consultations, and determinants of help-seeking behaviors. Sleep Med.

[B45] Motaghinejad M, Motevalian M, Fatima S, Faraji F, Mozaffari S (2017). The neuroprotective effect of curcumin against nicotine-induced neurotoxicity is mediated by CREB–BDNF signaling pathway. Neurochem Res.

[B46] Nam GE, Han K, Lee G (2017). Association between sleep duration and menstrual cycle irregularity in Korean female adolescents. Sleep Med.

[B47] Noorafshan A, Karimi F, Kamali AM, Karbalay-Doust S, Nami M (2017a). Restorative effects of curcumin on sleep-deprivation induced memory impairments and structural changes of the hippocampus in a rat model. Life Sci.

[B48] Noorafshan A, Karimi F, Karbalay-Doust S, Kamali AM (2017b). Using curcumin to prevent structural and behavioral changes of medial prefrontal cortex induced by sleep deprivation in rats. EXCLI J.

[B49] Pallesen S, Sivertsen B, Nordhus IH, Bjorvatn B (2014). A 10-year trend of insomnia prevalence in the adult Norwegian population. Sleep Med.

[B50] Parry BL, Hauger R, LeVeau B, Mostofi N, Cover H, Clopton P, Gillin JC (1996). Circadian rhythms of prolactin and thyroid-stimulating hormone during the menstrual cycle and early versus late sleep deprivation in premenstrual dysphoric disorder. Psychiatry Res.

[B51] Parsamanesh N, Moossavi M, Bahrami A, Butler AE, Sahebkar A (2018). Therapeutic potential of curcumin in diabetic complications. Pharmacol Res.

[B52] Saberi-Karimian M, Ghazizadeh H, Mohammadzadeh E, Ferns GA, Ghayour-Mobarhan M, Sahebkar A (2021). Does curcumin have an effect on sleep duration in metabolic syndrome patients?. Avicenna J Phytomed.

[B53] Shafabakhsh R, Mobini M, Raygan F, Aghadavod E, Ostadmohammadi V, Amirani E, Mansournia MA, Asemi Z (2020a). Curcumin administration and the effects on psychological status and markers of inflammation and oxidative damage in patients with type 2 diabetes and coronary heart disease. Clin Nutr ESPEN.

[B54] Shechter A, Lespérance P, Kin NNY, Boivin DB (2012). Nocturnal polysomnographic sleep across the menstrual cycle in premenstrual dysphoric disorder. Sleep Med.

[B55] Shoba G, Joy D, Joseph T, Rajendran MMR, Srinivas P (1998). Influence of piperine on the pharmacokinetics of curcumin in animals and human volunteers. Planta Med.

[B56] Srinivasan K (2007). Black pepper and its pungent principle-piperine: a review of diverse physiological effects. Crit Rev Food Sci Nutr.

[B57] Steiner M, Macdougall M, Brown E (2003). The premenstrual symptoms screening tool (PSST) for clinicians. Arch Womens Ment Health.

[B58] Szmidt MK, Granda D, Sicinska E, Kaluza J (2020). Primary dysmenorrhea in relation to oxidative stress and antioxidant status: A systematic review of case-control studies. Antioxidants.

[B59] Vaghela N, Mishra D, Sheth M, Dani VB (2019). To compare the effects of aerobic exercise and yoga on Premenstrual syndrome. J Educ Health Promot.

[B60] Wang Y, Gu F, Deng M, Guo L, Lu C, Zhou C, Chen S, Xu Y (2016). Rotating shift work and menstrual characteristics in a cohort of Chinese nurses. BMC women's health.

[B61] Whelan AM, Jurgens TM, Naylor H (2009). Herbs, vitamins and minerals in the treatment of premenstrual syndrome: a systematic review. Can J Clin Pharmacol.

[B62] Wichianpitaya J, Taneepanichskul S (2013). A comparative efficacy of low-dose combined oral contraceptives containing desogestrel and drospirenone in premenstrual symptoms. Obstet Gynecol Int.

[B63] Xing X, Xue P, Li SX, Zhou J, Tang X (2020). Sleep disturbance is associated with an increased risk of menstrual problems in female Chinese university students. Sleep Breath.

[B64] Yazdi Z, Sadeghniiat-Haghighi K, Zohal MA, Elmizadeh K (2012). Validity and reliability of the Iranian version of the insomnia severity index. Malays J Med Sci.

[B65] Young TB (2004). Epidemiology of daytime sleepiness: definitions, symptomatology, and prevalence. J Clin Psychiatry.

[B66] Yu M, Han K, Nam GE (2017). The association between mental health problems and menstrual cycle irregularity among adolescent Korean girls. J Affect Disord.

